# SARS‐CoV‐2‐Induced Macrophage Polarization Reverses HIV‐1 Latency in J‐Lat Cells Through TNFα Signaling

**DOI:** 10.1155/jimr/9986845

**Published:** 2026-02-25

**Authors:** Patricio Jarmoluk, Franco Agustín Sviercz, Cintia Cevallos, Rosa Nicole Freiberger, Cynthia Alicia López, M. Victoria Delpino, Jorge Quarleri

**Affiliations:** ^1^ Viral Immunopathology Laboratory, Institute for Biomedical Research on Retroviruses and AIDS (INBIRS), National Scientific and Technical Research Council (CONICET), University of Buenos Aires (UBA), Buenos Aires, Argentina, uba.ar

**Keywords:** COVID-19, HIV latency, J-Lat, macrophage polarization, ROS, SARS-CoV-2, TNFα

## Abstract

**Introduction:**

Coronavirus disease 2019 (COVID‐19) may have both short‐ and long‐term impacts on the progression of human immunodeficiency virus (HIV)‐1 after acute SARS‐CoV‐2 infection in people living with HIV (PLWH), even those on combined antiretroviral therapy (cART). This study aimed to investigate whether SARS‐CoV‐2 could influence HIV reactivation in latently infected lymphoid cells.

**Methods:**

HIV‐infected lymphoid (J‐Lat) cells, characterized by proviral latency under unstimulated conditions, were used for latency reversal assays with phorbol 12‐myristate 13‐acetate (PMA), free SARS‐CoV‐2 particles, or conditioned media (CM) from macrophages. Monocytes isolated from donor blood were differentiated into macrophages (monocyte‐derived macrophages [MDMs]) and polarized to M1 or M2 phenotypes before stimulation or with two SARS‐CoV‐2 variants (wild‐type and BA.5) infection. Additionally, the effects of redox imbalance on latency reversal in both J‐Lat and myeloid (U1) latency models were measured. SARS‐CoV‐2 RNA was quantified by RT‐qPCR targeting ORF1ab and N genes, and latency reversal and reactive oxygen species (ROS) levels were assessed by flow cytometry. TNFα involvement was confirmed through neutralization assays, while cytokines and polarization markers were analyzed via ELISA and fluorescent antibodies.

**Results:**

Jurkat and J‐Lat cells had low ACE2 expression and were not permissive to SARS‐CoV‐2 infection. SARS‐CoV‐2 exposure alone did not induce HIV latency reversal in J‐Lat cells. However, CM from M1‐polarized, resiquimod (R‐848)‐treated, and SARS‐CoV‐2‐infected macrophages significantly reactivated latent HIV. TNFα was identified as the primary driver of latency reversal, with no significant changes in ROS levels. Prolonged SARS‐CoV‐2 exposure shifted macrophage polarization toward an anti‐inflammatory M2 phenotype, characterized by IL‐10 release, which reduced latency reactivation.

**Conclusions:**

This study demonstrates that SARS‐CoV‐2 can indirectly reverse HIV latency in lymphoid cells by promoting the release of pro‐inflammatory cytokines from infected macrophages. These findings suggest potential therapeutic strategies for preventing HIV reactivation during SARS‐CoV‐2 coinfection, emphasizing the modulation of cytokine signaling to control inflammation while minimizing immune dysregulation.

## 1. Introduction

A significant challenge in finding a cure for human immunodeficiency virus (HIV) is the existence of a diverse reservoir of infected cells that continue to survive even with combined antiretroviral therapy (cART) [1, 2]. When cART is stopped, these latently infected cells can become active again, resulting in viral production and a swift increase in viremia [3]. Among these cells, latently infected CD4^+^ T cells form the largest and most stable reservoir, posing a major obstacle to HIV eradication [4, 5]. Despite extensive studies, the effects of SARS‐CoV‐2 infection on the HIV reservoir in people living with HIV (PLWH) are still not well understood.

Monocyte‐derived macrophages (MDMs) show remarkable adaptability in tissues, responding to local cytokine environments and differentiating into specialized types, such as M1 pro‐inflammatory or M2 anti‐inflammatory macrophages. The differentiation and function of MDMs are shaped by various stimuli, including highly pro‐inflammatory microenvironments, which can trigger HIV reactivation from latency in other cell types, potentially creating new reservoirs [6, 7]. A key aspect of SARS‐CoV‐2 infection is the strong inflammatory response seen in severe coronavirus disease 2019 (COVID‐19) cases, marked by an overwhelming release of pro‐inflammatory cytokines—like TNFα, IL‐1β, and IL‐6—often referred to as a “cytokine storm.” Furthermore, macrophages infected with SARS‐CoV‐2 tend to adopt a mainly pro‐inflammatory M1‐polarized phenotype early in the infection [8–10]. This inflammatory environment raises the question of whether SARS‐CoV‐2 infection can disrupt proviral latency in PLWH, either directly or indirectly. In this regard, we have recently shown that exposing MDMs to SARS‐CoV‐2 initially drives their polarization toward a pro‐inflammatory M1 phenotype, which later shifts to an anti‐inflammatory M2 phenotype over time.

This dynamic polarization was associated with the release of soluble factors during the early M1 phase, which reactivated HIV production in persistently infected promonocytic cells (U1), similar to the effects seen in MDMs stimulated with the TLR7/8 agonist resiquimod (R‐848) [11]. Additionally, there are cell‐specific differences in how HIV latency is modulated, with redox imbalances in the cellular reduction status affecting virus production. The balance of cellular redox homeostasis plays a role in HIV transcription, indicating that MDMs manage HIV latency differently compared to T cells and monocytes [12]. This complexity highlights the intricate relationship between the cellular environment and the regulation of HIV latency, emphasizing the importance of studying these dynamics in the context of SARS‐CoV‐2 infection.

To address these knowledge gaps, we utilized J‐Lat cells, a well‐established in vitro lymphoid model of HIV latency [13], to investigate the potential effects of SARS‐CoV‐2 on proviral reactivation. Through a combined virological, immunological, and biochemical approach, we evaluated whether exposure to free SARS‐CoV‐2 or soluble factors released from infected MDMs could influence HIV latency. Furthermore, we examined the impact of redox imbalance on latency reactivation by analyzing the effects of reactive oxygen species (ROS) inducers and inhibitors in both J‐Lat lymphoid cells and myeloid‐derived U1 promonocytic cells. The methodologies employed here offer a comprehensive understanding of the complex interplay between SARS‐CoV‐2 infection and HIV latency.

## 2. Materials and Methods

### 2.1. Cell Lines, Primary Cells, and Reagents

The J‐Lat 10.6 lymphocytic cell line was obtained from the AIDS Reagent Program of the NIH, USA [14, 15]. J‐Lat 10.6, a GFP‐expressing Jurkat cell line with a full‐length HIV‐1 genome but a nonfunctional Env., contains one copy of the virus per cell [16]. The uninfected Jurkat cell line was also used. Both cell lines were maintained in RPMI complete medium (Gibco, USA), including 10% fetal bovine serum (FBS, Sigma‐Aldrich, Argentina), 2 mM L‐glutamine (Gibco), 1 mM sodium pyruvate (Gibco), and penicillin–streptomycin (Sigma), at 37°C and 5% CO_2_. Mycoplasma contamination was routinely tested using the MycoAlert Mycoplasma Detection Kit (Lonza, USA). J‐Lat cells were seeded at 1 × 10^5^ cells/mL in 24‐well plates and divided into three groups as follows: (1) unstimulated control (negative control), (2) stimulated with phorbol 12‐myristate 13‐acetate (PMA), 30 ng/mL (Merck, USA) (positive control), and (3) exposed to different candidate stimuli. After 48 h of incubation, cells were centrifuged at 300 *g* for 4 min and resuspended in 100 μL of phosphate‐buffered saline (PBS). HIV reactivation was measured by GFP fluorescence intensity using a Cytek Northern Lights 3000 (Cytek Biosciences, USA) flow cytometer.

Myeloid U1 cells were also obtained from the AIDS Reagent Program of the NIH, as previously described [11]. These cells—originally cloned from promonocytic U937 cells surviving acute infection with the HIV‐1 LAI/IIIB strain—were seeded at 1 × 10^5^ cells/mL in 24‐well plates and maintained in RPMI complete medium (Gibco) in an incubator at 37°C and 5% CO_2_ atmosphere.

All experiments adhered to BSL‐3 laboratory standards at INBIRS, with biological materials autoclaved and incinerated following institutional rules.

### 2.2. Primary Monocytes and Macrophages

Human monocytes were isolated from the blood of healthy donors and differentiated intoMDMs as we described previously [11, 17, 18]. Briefly, monocytes were seeded at 5 × 10^5^ cells/mL in RPMI complete medium and 10 ng/mL M‐CSF (StemCell Technologies, Canada) for 6 days. Differentiated cells were confirmed to be >90% CD68^+^ by flow cytometry. MDMs were either left unstimulated (M0) or polarized for 24 h with either LPS (100 ng/mL) (Invivogen, USA) and IFN‐γ (20 ng/mL) (R&D, USA) for M1 or IL‐4 (20 ng/mL) (R&D) for M2 polarization.

MDMs were stimulated with the TLR 7/8 agonist resiquimod (1 μg/mL, R‐848, Alexis) at 37°C, 5% CO_2_ for 24 h, as well.

Supernatants (named as conditioned media [CM]) were collected and stored at −80°C until testing on J‐Lat cells.

### 2.3. SARS‐CoV‐2 Infection and RNA Quantification

The SARS‐CoV‐2 wild‐type strain (Wh) and Omicron (BA.5) strain were characterized, propagated, and titrated in Vero E6/TMPRSS2 cells. SARS‐CoV‐2 genomic RNA was detected and quantified using the Chemagic Viral DNA/RNA kit on the Chemagic 360 instrument (PerkinElmer, Germany). After infection, supernatants were collected at T0 (after washing) and 72 hpi. RNA was normalized and detected by RT‐qPCR (DisCoVery SARS‐CoV‐2 RT‐PCR Detection Kit Rox, AP Biotech, Argentina) targeting ORF1ab and N genes.

MDMs (5 × 10^5^ cells/mL) were cultured in RPMI complete medium with 10 ng/mL M‐CSF for 3 days, then incubated with SARS‐CoV‐2 (Wh variant) at multiplicity of infection (MOI) = 0.1 for 24, 48, and 72 h (hpi). Supernatants (CM) were collected, centrifuged, and stored at −80°C.

### 2.4. HIV Latency Reversal Assays

J‐Lat (and U1 for redox experiments) cells were exposed to CM or treated with various chemical modulators of latency. These included PMA (30 ng/mL), R‐848 (1 μg/mL, Alexis), TNFα (1 ng/mL), and ROS inducers such as rotenone and tert‐butyl hydroperoxide (TBH), both tested at different concentrations. Treatments were applied for 48 h, after which cells were analyzed by flow cytometry to evaluate HIV reactivation.

For experiments involving N‐acetyl‐L‐cysteine (5 μM, NAC; Cayman Chemicals, USA), the compound was added 1.5 h before adding latency reversal agents and remained in the media. Flow cytometry analysis was performed at various time points posttreatment to evaluate reactivation dynamics.

### 2.5. Cellular ROS Measurements

Cellular ROS production, including hydroxyl, peroxyl, and other reactive oxygen species, was assessed using the 2′,7′‐dichlorofluorescein diacetate (DCFDA) assay kit (Abcam, United Kingdom) following the manufacturer’s instructions. The assay employs DCFDA, a cell‐permeable fluorogenic dye. Upon entry into cells, DCFDA is deacetylated by cellular esterases into a nonfluorescent intermediate, which ROS subsequently oxidizes to form DCF, a highly fluorescent compound. DCF fluorescence was detected using excitation and emission maxima of 495 and 529 nm, respectively.

At 48 h postexposure, J‐Lat and U1 cells were washed twice with PBS and incubated with 25 μM DCFDA in the essential medium at 37°C for 30 min. After incubation, ROS levels were quantified by flow cytometry.

Since mitochondria are both the initiation point for the intrinsic pathway of cell death activation and the primary source of ROS production in cells, we sought to elucidate their role in HIV‐latency reversal, as previously described [19]. Briefly, mitochondrial ROS (mROS) levels were assessed by flow cytometry in J‐Lat and U1 cells stained with 5 μM MitoSOX Red reagent (Thermo Fisher Scientific, USA) for 30 min. This assay utilizes a positively charged probe that rapidly accumulates in mitochondria. Once inside, the probe is oxidized by mitochondrial reactive species, including superoxide, to form ethidium, which binds to mitochondrial DNA and emits fluorescence detectable by flow cytometry.

### 2.6. Flow Cytometry Analysis

Data were acquired on a Cytek Northern Lights 3000 Flow Cytometer and analyzed with FlowJo.v10.6.2 (Ashland, USA).

MDMs were detached using Accutase (Stem Cell Technologies) and then phenotypically characterized by staining with anti‐human CD80 (PE, BioLegend, United Kingdom), CD206 (APC, BioLegend), CD16 (R688, Tonbo, Cytek Biosciences Inc., USA), and CD14 (FITC, BioLegend). After staining, samples were incubated at room temperature for 30 min in the dark and washed with 1 mL PBS containing 2% FBS.

Jurkat and J‐Lat cells were assessed for ACE2 expression using a rabbit primary polyclonal antibody (PA5.20040, Thermo Fisher Scientific) for 1 h on ice. The cells were washed and incubated with a PE‐labeled anti‐rabbit antibody (ab72465, Abcam).

Latency reversal among U1 cells was analyzed by HIV‐1 p24 Gag expression in fixed and permeabilized cells with the BD Cyto Fix/Perm kit (BD Biosciences, San Jose, CA, USA), followed by washing with PBS supplemented with 1% FBS. Staining was performed using the anti‐HIV‐1 p24 Gag phycoerythrin (PE) monoclonal antibody KC57 (Beckman Coulter, Brea, CA, USA) at a dilution of 1:250. Isotype‐matched antibodies served as negative controls for the assay.

Cell death was assessed with Ghost Dye Violet450 (Tonbo, Cytek Biosciences Inc., USA).

Gating strategies were performed using FlowJo v10.6.2. Cell populations were first gated based on forward and side scatter to exclude debris, followed by singlet gating (FSC‐A vs. FSC‐H) and exclusion of dead cells using Ghost Dye Violet450. For phenotypic analysis of macrophages, CD14^+^ cells were selected and further classified as M1 (CD80^+^CD206^−^) or M2 (CD80^−^CD206^+^). In J‐Lat and U1 cells, GFP expression or intracellular p24 staining, respectively, was used to assess HIV reactivation. A detailed schematic of the complete gating strategy is provided in Supporting Information 1: Figure 2.

### 2.7. ELISA for Cytokine Detection

TNFα, IL‐6, IL‐1β, and IL‐10 were measured in culture supernatants by sandwich ELISA (BD Pharmingen, USA).

### 2.8. TNFα Neutralization

CM from SARS‐CoV‐2‐exposed MDMs was preincubated with the TNFα‐neutralizing antibody infliximab (Chimeric Recombinant Human Monoclonal Antibody cA2, Cat# MA5‐47798, Thermo Fisher) at a concentration of 25 μg/mL for 2 h at 37°C before being added to J‐Lat cells. The mAb concentration required to neutralize TNFα was optimized by stimulating MDMs with recombinant TNFα (1 ng/mL) in the presence of varying antibody concentrations. J‐Lat cells were stimulated directly with human recombinant TNFα (1 ng/mL) as control.

### 2.9. Statistical Analysis

Data were analyzed using GraphPad Prism 9.4 (GraphPad Software Inc., USA). Normal distribution datasets used paired or unpaired *t*‐tests, while nonnormal datasets used Mann–Whitney or Wilcoxon tests. Significance was set at *p* < 0.05 and represented in each figure when it corresponds ( ^∗^ *p* ≤ 0.05;  ^∗∗^ *p* ≤ 0.01;  ^∗∗∗^ *p* ≤ 0.001; and  ^∗∗∗∗^ *p* ≤ 0.0001).

## 3. Results

### 3.1. Low ACE2 Surface Expression and Lack of SARS‐CoV‐2 Permissiveness in Jurkat and J‐Lat Cells

Flow cytometry analysis showed that only a small percentage of unstimulated J‐Lat cells had detectable levels of ACE2, the receptor for SARS‐CoV‐2, with a mean expression of 2.8% ± 0.7% (Figure 1A). This was similar to the levels found in unstimulated Jurkat cells, which had an expression of 5.1% ± 1.0%. After a 48‐h incubation with CM from uninfected MDM, SARS‐CoV‐2‐exposed MDM, or M2‐polarized MDM, ACE2 expression remained stable at 2.9% ± 1.4%, 6.1% ± 1.1%, and 4.1% ± 2.6%, respectively. No significant changes in ACE2 expression were noted after exposure to CM from M1‐polarized MDM (3.9% ± 0.9%) or cell‐free SARS‐CoV‐2 (3.3% ± 0.3%). However, PMA stimulation of J‐Lat cells did lead to a modest increase in ACE2 expression (12.3% ± 3.0%) (Figure 1A). To assess the capacity of J‐Lat and Jurkat cells to support productive SARS‐CoV‐2 infection, the cells were exposed to the wild‐type (Wh) variant at an MOI of 0.1. Viral RNA levels for the N and ORF‐1a genes were measured in the culture supernatants using RT‐qPCR. The results indicated a gradual decrease in viral RNA levels over 72 h across all tested conditions (Figure 1B). These results confirm that Jurkat and J‐Lat cells are not permissive to SARS‐CoV‐2 infection and show similarly low surface expression of ACE2.

Figure 1Level of ACE2 expression in J‐Lat and Jurkat (JK) cells, and kinetics of the SARS‐CoV‐2 replication. (A) J‐Lat cells were exposed to different conditions, such as NT (nontreated); cfv (cell‐free SARS‐CoV‐2, MOI: 0.1); MDM‐CM (conditioned media) obtained from noninfected macrophages (Ni), exposed to SARS‐CoV‐2 (Wh), and polarized (M1or M2, diluted 1:100). PMA: positive control for reversal J‐Lat cells (*n* = 3). (B) Kinetics of the SARS‐CoV‐2 (Wh variant, MOI: 0.1) replication in lymphoid (J‐Lat, Jurkat) cells measured using RT‐qPCR targeted to N and ORF1a genes in the culture supernatant, as described in M&M. T0: supernatant obtained after cell washing; 72 h: 3 days postinfection (*n* = 3). Data are presented as the mean ± SD. (A and B) *n* = 3 independent experiments; each experiment was assayed in technical duplicate, and statistical significance was calculated using one‐way ANOVA.  ^∗^ *p* < 0.05;  ^∗∗∗^ *p* < 0.001; and  ^∗∗∗∗^ *p* < 0.0001. Each independent experiment corresponds to one donor.

**Figure:**
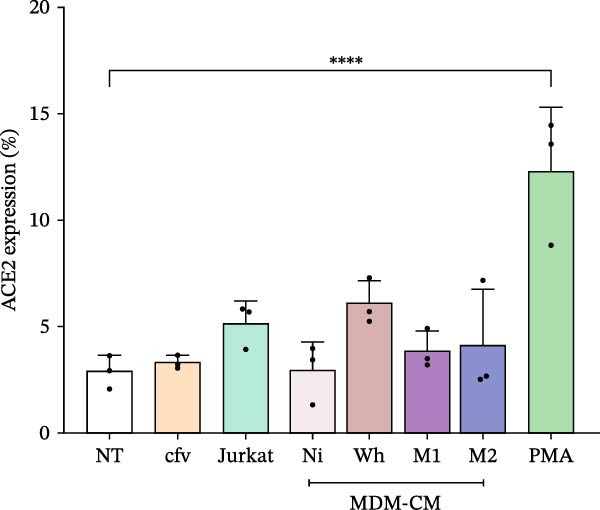
(A)

**Figure:**
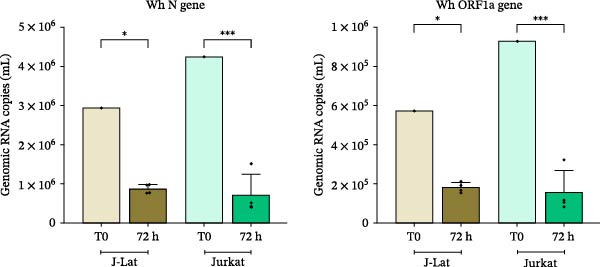
(B)

### 3.2. Cell‐Free SARS‐CoV‐2 Does Not Induce HIV‐1 Latency Reversal in J‐Lat Cells

The percentage of reactivation among J‐Lat cells that were exposed to cell‐free SARS‐CoV‐2 for 4 h, at both high (MOI = 1) and low (MOI = 0.1) multiplicities of infection, did not show any significant differences when compared to control cells (NT: 1.2% ± 0.3%, Wh (0.1): 1.1% ± 0.3%, BA.5 (0.1): 0.9% ± 0.1%, Wh (1.0): 0.9% ± 0.1%, BA.5 (1.0): 0.9% ± 0.1%) (Figure 2A). Likewise, as illustrated in Figure 2B, there were no significant differences noted after 24 h (NT: 0.7 ± 0.2%, Wh (0.1): 0.7% ± 0.2%, BA.5 (0.1): 0.7% ± 0.1%), 48 h (NT: 0.8% ± 0.1%, Wh (0.1): 0.7% ± 0.1%, BA.5 (0.1): 0.8% ± 0.04%), or 72 h (NT: 0.8% ± 0.1%, Wh (0.1): 0.8% ± 0.1%, BA.5 (0.1): 0.8% ± 0.1%) of exposure to cell‐free SARS‐CoV‐2. These results suggest that cell‐free SARS‐CoV‐2 particles do not directly activate proviral HIV transcription through cell signaling pathways in lymphocytic cells.

Figure 2HIV‐latency reversal in the J‐Lat lymphoid model. (A) Level of HIV‐latency reversal in lymphoid model exposed to cell‐free SARS‐CoV‐2 during 4 h, two variants (Wh and BA.5), and two inoculums (MOI: 0.1 and 1.0) (A1, *n* = 4), and representative dot plots of flow cytometry data (A2). (B) Level of HIV‐latency reversal in lymphoid model exposed to cell‐free SARS‐CoV‐2 (MOI: 0.1), during extended times (24, 48, and 72 h), with Wh and BA.5 variants (B1, *n* = 5), and representative dot plots obtained by flow cytometry for each condition (B2). NT: nontreated cells. PMA was used as a positive control for HIV latency reversal. Data are presented as the mean ± SD. (A) *n* = 4; (B) *n* = 5 independent experiments; each experiment was assayed in technical duplicate, and statistical significance was calculated using one‐way ANOVA. Each independent experiment corresponds to one donor.

**Figure:**
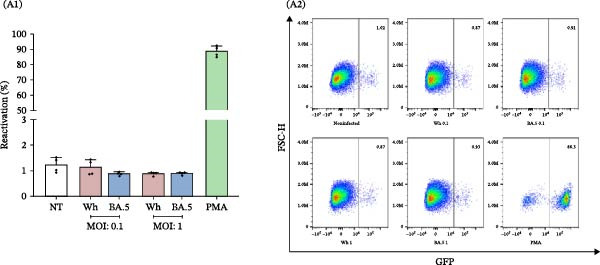
(A)

**Figure:**
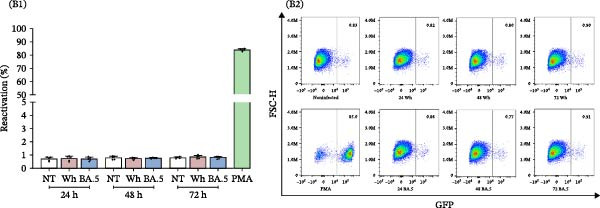
(B)

### 3.3. HIV Latency Modulation by Cytokines From Polarized Macrophages

Primary human MDMs were polarized toward M1 and M2 phenotypes using IFN‐γ plus LPS and IL‐4, respectively, following established protocols [11, 20]. The expression of membrane markers CD80 and CD206 was evaluated by flow cytometry, while cytokine release (IL‐1β, TNFα, IL‐6, and IL‐10) was quantified using ELISA.

M1‐MDMs exhibited significantly elevated CD80 expression (83.2% ± 5.5%) compared to control (M0:2.6% ± 1.1%) and M2‐MDMs (3.4% ± 1.4%), whereas CD206 expression was markedly higher in M2‐MDMs (89.8% ± 3.7%). Interestingly, 42.2% ± 4.8% of M1‐MDMs retained CD206 expression levels similar to unpolarized cells, indicating heterogeneity within the M1 population.

Cytokine concentrations in the CM were as follows: M0‐MDM: 3.3 ± 2.6 pg/mL (IL‐1β), 0.4 ± 0.3 ng/mL (TNFα), 1.2 ± 0.6 ng/mL (IL‐6), and 11.2 ± 2.2 pg/mL (IL‐10); M1‐MDM: 281.4 ± 90.0 pg/mL (IL‐1β), 27.4 ± 11.1 ng/mL (TNFα), 6.4 ± 2.4 ng/mL (IL‐6), and 38.1 ± 5.7 pg/mL (IL‐10); M2‐MDM: 55.1 ± 16.3 pg/mL (IL‐1β), 1.3 ± 0.4 ng/mL (TNFα), 1.1 ± 0.3 ng/mL (IL‐6), and 553.1 ± 87.6 pg/mL (IL‐10) (Figure 3A, B).

Figure 3Characterization and profiles of human monocyte‐derived primary macrophages and their capability for HIV‐latency reversal in the J‐Lat model. (A) Characterization of primary human macrophages polarized to M1 and M2 using the membrane expression level of CD80 and CD206 (A1, *n* = 4) and representative dot plots of flow cytometry data (A2). Single‐marker frequencies are shown, and mutually exclusive M1/M2 subsets were defined using the CD80 vs CD206 quadrant gating (CD80^+^CD206^−^ = M1; CD206^+^CD80^−^ = M2). (B) The levels of cytokines IL‐1β, IL‐6, TNFα, and IL‐10 in culture supernatants were quantified by ELISA. These cell culture supernatants from each were collected to be used as “conditioned media” (*n* = 6). (C) Level of HIV‐latency reversal in J‐Lat cells exposed to conditioned media (diluted as indicated) from M1‐polarized macrophages (C1, *n* = 4) and representative dot plots of flow cytometry data (C2). (D) Level of HIV‐latency reversal in J‐Lat cells exposed to conditioned media (diluted as indicated) from M2‐polarized macrophages (lD1, *n* = 4) and representative dot plots of flow cytometry data (D2). M0: unpolarized MDMs; CM‐RSQ: CM obtained from MDMs treated with R‐848; RSQ: J‐Lat treatment directly with R‐848. PMA was used as a positive control for HIV latency reversal. Data are presented as the mean ± SD. (A, C, and D) *n* = 4; (B) *n* = 6 independent experiments; each experiment was assayed in technical duplicate, and statistical significance was calculated using one‐way ANOVA.  ^∗∗∗^ *p* < 0.001 and  ^∗∗∗∗^ *p* < 0.0001. Each independent experiment corresponds to one donor.

**Figure:**
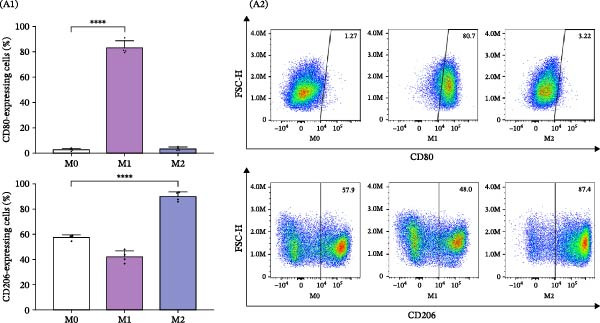
(A)

**Figure:**
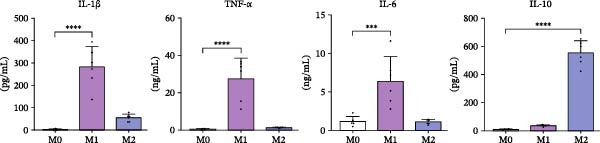
(B)

**Figure:**
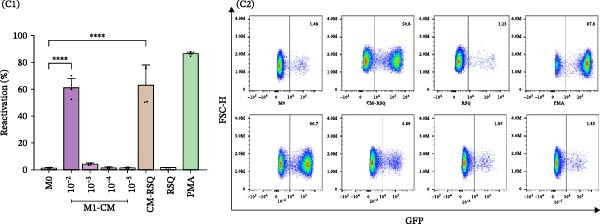
(C)

**Figure:**
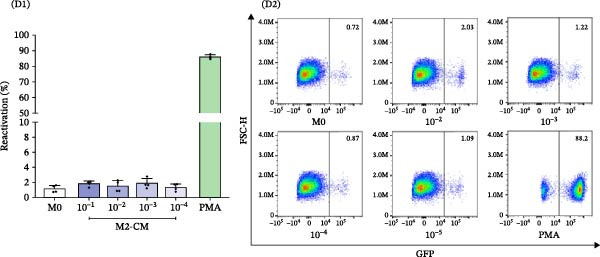
(D)

To investigate the effects of CM on HIV latency, J‐Lat cells were treated with CM from unpolarized (M0) or M1/M2‐polarized MDMs. PMA‐stimulated cells served as a positive control (86.1 ± 1.3%). CM from M1‐MDMs collected 24 h post‐polarization induced significant, concentration‐dependent latency reversal, with reactivation levels of 60.8 ± 7.1% and 4.1 ± 0.7% at 1:100 and 1:1000 dilutions, respectively. Conversely, the CM from M2‐MDMs failed to reactivate the latent virus.

Consistent with previous reports [21], when J‐Lat cells were exposed to CM from control MDMs treated with the TLR7/8 agonist R‐848 for 48 h—known to repolarize M2 macrophages to an M1 phenotype [22]—a substantial proportion of GFP‐positive cells were detected (63.1% ± 14.7%). Notably, this latency reversal was not observed when J‐Lat cells were directly exposed to the agonist (1.3% ± 0.1%) (Figure 3C, D).

These results indicate that soluble factors released by M1‐MDMs and R‐848‐treated MDMs can reactivate latent HIV‐1 in J‐Lat cells. Notably, this latency reversal occurs indirectly through soluble factors rather than through direct activation of endosomal TLR7/8 receptors by the virus.

### 3.4. Phenotypic Characterization of SARS‐CoV‐2‐Infected Macrophages Capable of Reversing HIV Latency in J‐Lat Cells

The study of macrophage polarization dynamics after long‐term exposure (24, 48, and 72 h) to SARS‐CoV‐2 (Wh variant) revealed changes in the M1/M2 relative abundance based on the duration of virus exposure (Figure 4A). The proportions of M1 and M2 macrophages varied over time with the virus. Initially, M1 macrophages (CD80^+^) were predominant, making up 75.5% ± 9.2% at 24 h. However, their numbers significantly dropped to 68.1% ± 11.5% at 48 h and further to 51.6% ± 7.4% at 72 h. Conversely, M2 macrophages (CD206^+^) exhibited a consistent increase in relative abundance (%), starting from levels similar to control cells at 24 h (63.9 ± 9.8), rising to 81.6 ± 6.2 at 48 h, and reaching a peak of 92.2 ± 2.1 at 72 h.

Figure 4Polarization of primary human macrophages (MDMs) exposed to SARS‐CoV‐2 during extended times (24, 48, and 72 h). (A) Level of CD80 and CD206 membrane expression (A1, *n* = 6) and representative dot plots of flow cytometry data (A2). (B) Level of cytokines IL‐1β, IL‐6, TNFα, and IL‐10 in culture supernatants (*n* = 8); these cell culture supernatants (A) and (B) were collected to be used as “conditioned media” (CM). (C) Level of HIV‐latency reversal in lymphoid model exposed for a fixed time (48 h) to CM collected at 24 and 72 h after SARS‐CoV‐2 exposure (C1, *n* = 13) and representative dot plots of flow cytometry data (C2). (D) MDMs membrane expression of CD14 and CD16 (D1, *n* = 10) and their representative histograms of median fluorescence intensity (MFI) for each condition (D2). Ni: noninfected; Wh: infected with the ancestral SARS‐CoV‐2 variant. Data are presented as the mean ± SD. (A) *n* = 4; (B) *n* = 8; (C) *n* = 13; (D) *n* = 10 independent experiments; each experiment was assayed in technical duplicate, and statistical significance was calculated using one‐way ANOVA.  ^∗^ *p* < 0.05;  ^∗∗^ *p* < 0.01;  ^∗∗∗^ *p* < 0.001; and  ^∗∗∗∗^ *p* < 0.0001. Each independent experiment corresponds to one donor.

**Figure:**
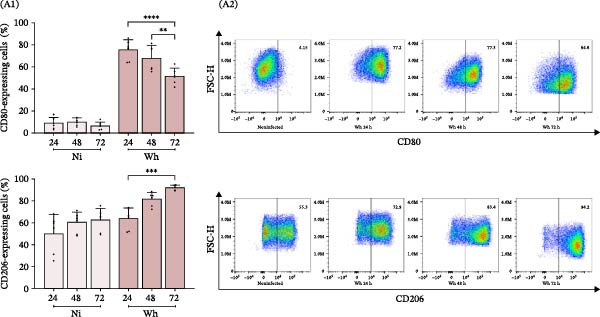
(A)

**Figure:**
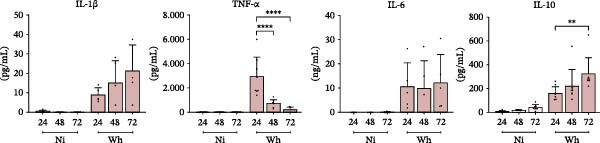
(B)

**Figure:**
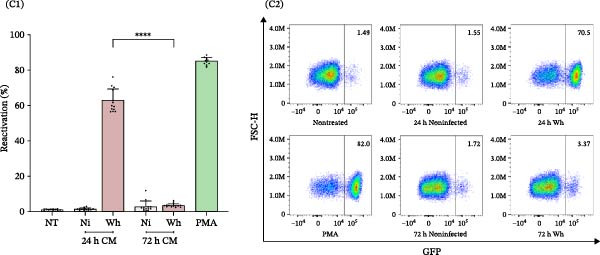
(C)

**Figure:**
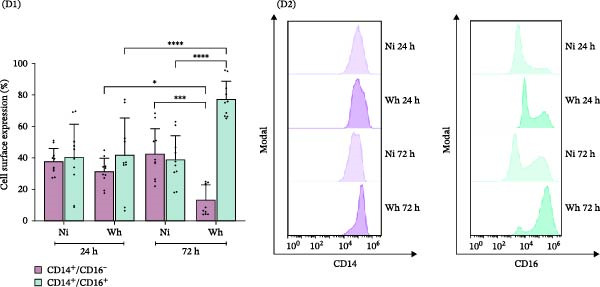
(D)

This rise in M2 macrophages was associated with higher levels of the anti‐inflammatory cytokine IL‐10 in the CM. The IL‐10 concentrations (pg/mL) from noninfected MDMs (10.1 ± 7.2, 18.9 ± 5.0, and 42.5 ± 24.1 at 24, 48, and 72 h, respectively) were significantly lower than those from SARS‐CoV‐2‐infected MDMs, which showed a substantial increase over the same time points (160.3 ± 56.7, 221.5 ± 138.9, and 324.5 ± 134.7, respectively) (Figure 4B).

Among the pro‐inflammatory cytokines, TNFα levels (pg/mL) in the CM reflected the M1 polarization profile (2937.0 ± 1603.0, 722.6 ± 331.9, and 221.5 ± 193.5 at 24, 48, and 72 h, respectively). Notably, the peak TNFα concentration in SARS‐CoV‐2‐infected MDMs was ten times lower than in M1 macrophage CM. IL‐1β levels (pg/mL) were relatively low but showed a gradual increase over time (8.9 ± 3.7, 15.1 ± 11.4, and 21.3 ± 13.5). In contrast, IL‐6 levels (ng/mL) did not show significant differences over the same period (10.6 ± 9.9, 9.9 ± 11.5, and 12.4 ± 11.6).

As illustrated in Figure 4C, only the CM collected 24 h after SARS‐CoV‐2 infection significantly reactivated HIV latency in J‐Lat cells, showing a reactivation rate of 54.4% ± 14.4%. In contrast, CM obtained at 72 h did not induce reactivation (2.5% ± 1.6%).

Given that the minor subset of CD14^+^CD16^+^ monocytes constitutes only 10% of the total monocyte population and plays an important role in TNFα production [23], we aimed to explore their response to SARS‐CoV‐2 infection. Noting the increase in IL‐10 levels following infection and understanding IL‐10’s role in driving macrophage differentiation—especially in enhancing the expression of Fc receptors like FcγRIII (CD16) [24]—we examined how SARS‐CoV‐2 affects the expression of these receptors in MDMs.

As depicted in Figure 4D, at 24 hpi, the relative abundance (%) of SARS‐CoV‐2‐infected CD14^+^/CD16^+^ MDMs was not significantly different from that of noninfected MDMs (41.7 ± 23.6 and 40.4 ± 21.0, respectively). However, by 3 dpi, the proportion of CD14^+^/CD16^+^ MDMs was significantly higher in the infected cells compared to the controls (77.4 ± 11.4 and 38.6 ± 15.4, respectively). This increase was accompanied by a notable decrease in the proportion of classical CD14^+^/CD16^−^ MDMs (13.1 ± 9.7 and 42.3 ± 16.3, respectively).

These results indicate that the reversal of HIV latency in J‐Lat cells occurs as a bystander effect driven by soluble factors released by macrophages exposed to SARS‐CoV‐2 for 24–72 h. The significant latency reversal observed within the first 24 h may be due to temporarily elevated levels of pro‐inflammatory cytokines, particularly TNFα, which likely exceed the threshold needed to disrupt latency mechanisms. However, extended exposure to SARS‐CoV‐2 leads to a shift in macrophage polarization toward an anti‐inflammatory M2 profile, characterized by an increased proportion of CD14+/CD16+ nonclassical subsets that release higher levels of IL‐10. This shift reduces the availability of activating signals, thereby diminishing HIV latency reversal.

### 3.5. TNFα Released by SARS‐CoV‐2‐Infected Macrophages Plays a Pivotal Role in HIV Latency Reversal in J‐Lat Cells

TNFα, a crucial pro‐inflammatory cytokine produced by M1 macrophages, is a strong candidate for latency reversal due to its established role in activating NF‐κB signaling pathways, which are essential for initiating viral transcription in latent cells [25]. To delve deeper into this, we examined the dose‐dependent effects of TNFα on proviral latency reversal in J‐Lat cells, taking into account its physiological serum levels [26].

As illustrated in Figure 5A, decreasing concentrations of TNFα (10, 1, and 0.1 ng/mL) led to a corresponding decline in latency reactivation (%), with values of 89.2% ± 2.1%, 66.5% ± 1.6%, and 14.2% ± 1.0%, respectively. Importantly, cell viability remained stable across all conditions, with viability percentages of 7.8% ± 1.4%, 2.3% ± 0.5%, and 0.8% ± 0.1%, respectively (Supporting Information 2: Figure S1).

Figure 5TNFα as a pivotal soluble mediator involved in HIV‐latency reversal. (A) TNFα‐mediated reversal of HIV latency in J‐Lat cells. Dose‐response relationship between TNFα concentrations (10, 1, and 0.1 ng/mL) and the percentage of latency reversal (% reactivation) (A1, *n* = 3), and representative dot plots of flow cytometry data (A2). (B) Neutralizing TNFα with infliximab (25 μg/mL) significantly reduces the latency reversal capacity of conditioned media from SARS‐CoV‐2‐infected MDMs (B1, *n* = 12), and representative dot plots of flow cytometry data (B2). NT: nontreated; Ni: noninfected. Data are presented as the mean ± SD. (A) *n* = 3; (B) *n* = 12 independent experiments; each experiment was assayed in technical duplicate, and statistical significance was calculated using one‐way ANOVA.  ^∗^ *p* < 0.05 and  ^∗∗∗^ *p* < 0.001. Each independent experiment corresponds to one donor.

**Figure:**
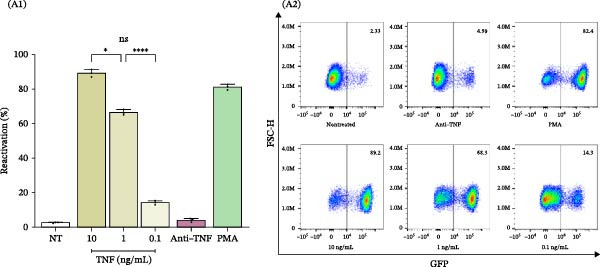
(A)

**Figure:**
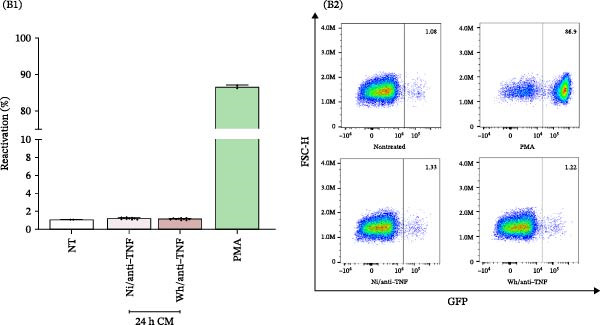
(B)

By using infliximab, a monoclonal antibody that neutralizes TNFα, we confirmed the critical role of this cytokine in HIV latency reversal. As shown in Figure 5B, the neutralization of TNFα diminished the reactivation capacity of CM media from SARS‐CoV‐2‐infected MDMs by 97.6% (from 54.4% ± 14.4% to 1.1% ± 0.0%). This provides direct evidence that TNFα is a significant driver of proviral activation.

Notably, the increased levels of TNFα found in SARS‐CoV‐2‐infected macrophages connect the inflammatory environment triggered by viral infection to latent HIV reactivation. The maintenance of cell viability in the neutralization assay further emphasizes the specificity of TNFα’s effect, ruling out nonspecific cytotoxic mechanisms. These findings underscore the therapeutic potential of targeting TNFα signaling pathways to influence HIV latency, especially in inflammatory contexts such as coinfections with SARS‐CoV‐2.

### 3.6. HIV Latency Reversal in J‐Lat and U1 Cells Is Independent of Redox Status Imbalance

MDMs are recognized for their sensitivity to changes in cellular redox status, using ROS to enhance their antimicrobial functions [27]. In the J‐Lat model of HIV latency, small‐molecule inhibitors that target proteins involved in redox homeostasis have been identified as latency‐promoting agents (LPAs) [28]. With this in mind, we aimed to explore whether TNFα’s role in regulating HIV latency in J‐Lat cells is linked to disruptions in cellular redox balance.

As illustrated in Figure 6A, J‐Lat cells treated with CM from SARS‐CoV‐2‐infected macrophages (Wh variant) for 1, 4, 8, 24, and 48 h exhibited no significant changes in mROS levels (% mROS‐producing cells: 1.5 ± 0.2, 0.8 ± 0.2, 1.7 ± 0.2, 1.1 ± 0.3, and 3.1 ± 2.2, respectively). In contrast, HIV reactivation showed a progressive increase (% GFP‐positive cells: 1.5 ± 0.2, 2.3 ± 0.5, 13.1 ± 1.1, 51.3 ± 2.7, and 75.6 ± 7.0, respectively).

Figure 6HIV latency reversal in J‐Lat cells is independent of ROS dynamics. (A) Mitochondrial ROS (% mROS) levels and level of latency reactivation (% GFP‐positive cells) were measured in J‐Lat cells exposed to conditioned media from SARS‐CoV‐2‐infected macrophages over time (1, 4, 8, 24, and 48 h) (A1, *n* = 4). Representative dot plots of flow cytometry data (A2) showing J‐Lat cells expressing mROS (upper) and GFP‐HIV (lower). (B) HIV reactivation in J‐Lat cells (as mean fluorescence intensity –GFP MFI‐) and redox imbalance (as MFI of DCFDA, or mROS) when treated with ROS inducers (TBH, rotenone) at different concentrations (B1, *n* = 4), and their representative histograms of median fluorescence intensity (MFI) for each condition (B2). (C) HIV reactivation in U1 cells (as mean fluorescence intensity –GFP MFI‐) and redox imbalance (as MFI of DCFDA, or mROS) when treated with ROS inducers (TBH, rotenone) at different concentrations (C1, *n* = 4), and their representative histograms of median fluorescence intensity (MFI) for each condition (C2). NAC: N‐acetyl‐L‐cysteine. Data are presented as the mean ± SD. (A, B, and C) *n* = 4 independent experiments; each experiment was assayed in technical duplicate. Each independent experiment corresponds to one donor.

**Figure:**
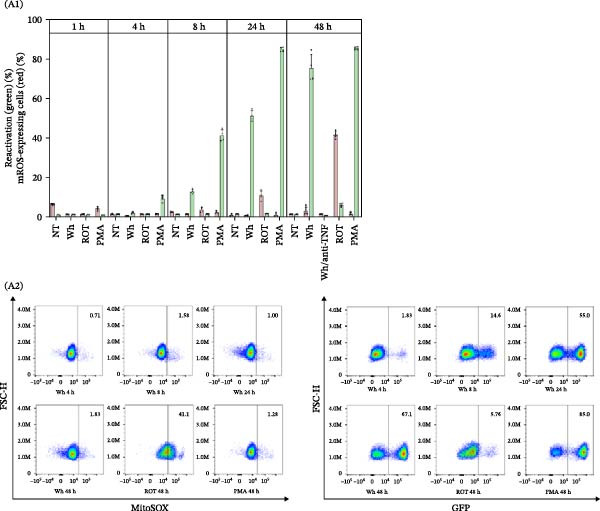
(A)

**Figure:**
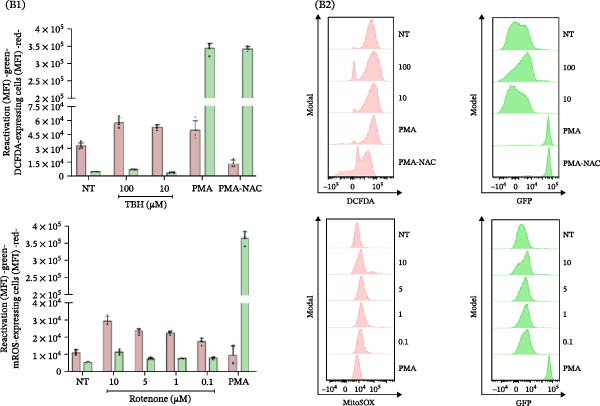
(B)

**Figure:**
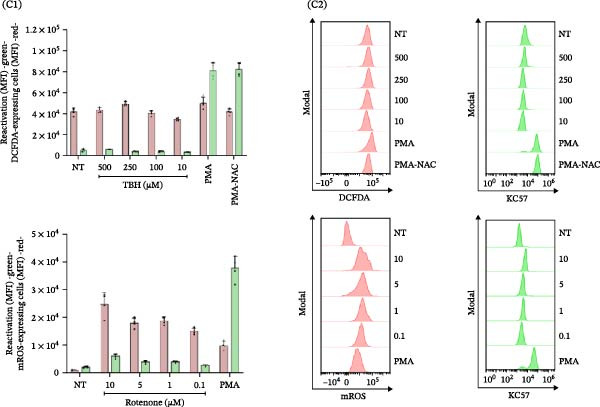
(C)

Similarly, latency reversal induced by PMA also increased over time (% GFP‐positive cells: 1.3 ± 0.0, 9.5 ± 1.7, 41.5 ± 2.3, 85.1 ± 1.1, and 85.6 ± 0.8, respectively) without significant changes in ROS levels. Neutralizing TNFα in the CM (from SARS‐CoV‐2‐infected MDMs) at 48 h did not alter mROS levels (1.7 ± 0.2%).

When J‐Lat cells were treated with rotenone (1 μM), which stimulates ROS production (% mROS: 1.6 ± 0.3, 1.7 ± 0.2, 3.9 ± 1.2, 11.0 ± 2.5, and 41.7 ± 1.9, respectively), no significant HIV reactivation was detected (% GFP‐positive cells: 1.3 ± 0.9 to 6.3 ± 1.9).

These findings indicate that HIV latency reversal in J‐Lat cells happens without relying on oxidative stress or changes in redox status, underscoring the unique mechanisms involved in latency reactivation within this model. Previous research has indicated that alterations in ROS‐induced NF‐κB activity, a pathway often associated with cellular activation, can facilitate HIV reactivation in macrophages, but not in T‐cells or monocytes [12]. To delve deeper into this, we examined whether inducing a redox imbalance (assessed through both DCFDA‐positive cells and mROS production) could trigger HIV latency reversal in J‐Lat and U1 cells (Figure 6B, C). The cells were treated for 1 h with increasing concentrations of rotenone (0.1, 1, 5, and 10 μM) and TBH (10, 100, 250, and 500 μM). However, neither rotenone nor TBH treatments led to significant proviral activation in either model, even at increasing concentrations. Similarly, when NAC, an antioxidant, was used in combination with PMA, the reduction in ROS levels did not affect the reactivation of latent HIV.

These findings emphasize that the reversal of HIV latency in lymphoid (J‐Lat) and monocytic (U1) models occurs independently of oxidative stress and ROS dynamics, showcasing different regulatory mechanisms when compared to macrophages.

## 4. Discussion

Both natural SARS‐CoV‐2 infections and mRNA vaccines present antigenic challenges that trigger strong humoral and cell‐mediated immune responses [29]. This initially raised concerns about their ability to activate HIV expression and promote viral release from latent reservoirs [30]. Theoretically, such activation could happen through the direct stimulation of reservoir cells that recognize vaccine antigens, including naïve CD4^+^ T‐cell reservoirs specific to SARS‐CoV‐2‐derived antigens [31, 32] or cross‐reactive memory CD4^+^ T‐cell reservoirs linked to common cold coronaviruses [33, 34]. Alternatively, broad inflammatory responses and cytokine production might temporarily activate HIV gene expression in nearby reservoir cells that do not directly respond to the antigen [35–37].

This study shows that ancestral (Wh) and Omicron (BA.5) SARS‐CoV‐2 variants, even without causing a productive infection, can reverse HIV latency in infected lymphoid cells through a bystander mechanism involving macrophages exposed to SARS‐CoV‐2. These macrophages release pro‐inflammatory cytokines, even in the absence of productive viral replication.

The main component of the HIV reservoir is made up of CD4^+^ T cells that contain integrated, replication‐competent provirus [38, 39]. Canonical NF‐κB activation plays a crucial role in driving HIV transcription and reversing latency [7]. Various in vitro models of HIV latency, such as J‐Lat cells, have been extensively utilized to investigate reservoir dynamics [25, 40, 41].

ACE2, the primary receptor for SARS‐CoV‐2, is an interferon‐stimulated gene (ISG) whose expression is linked to NF‐κB activity [42–44]. In line with previous findings, we noted low ACE2 expression in unstimulated Jurkat and J‐Lat cells. Additionally, SARS‐CoV‐2 did not produce detectable progeny or impact cell viability. However, recent research suggests that T lymphocytes, including Jurkat cells, can be infected by SARS‐CoV‐2 in an ACE2‐independent manner, resulting in significant T‐cell apoptosis in vitro [45, 46]. This inconsistency may arise from cellular activation before infection, which increases the likelihood of infection and apoptosis.

SARS‐CoV‐2 uses the CD4 receptor on T helper lymphocytes for infection [47], along with MHC class I/II and the TCR for activation [48]. The reduced CD4 expression [25] and the lack of MHC class I/II expression in leukemic T cells [49, 50] likely limit the direct interactions between the virus and the cells. Additionally, other potential receptors for SARS‐CoV‐2 in T lymphocytes have been suggested [51].

In this study, we provide an initial characterization of macrophage responses to SARS‐CoV‐2 using the well‐established surface markers CD80 (M1) and CD206 (M2) in an in vitro model. Our research indicates that M1 macrophages, unlike M0 or M2 macrophages, produce significantly higher levels of pro‐inflammatory cytokines, which can reverse HIV dormancy in J‐Lat cells. Among these cytokines, TNFα was found to be particularly abundant, along with IL‐6 and IL‐1β. Together, these cytokines foster a pro‐inflammatory environment that supports latency reversal [15, 52]. TNFα is known to strongly activate HIV‐1 transcription through NF‐κB binding sites in the LTR promoter [53] and has been extensively used in latency reversal assays involving J‐Lat cells. Similarly, IL‐1β and IL‐6 enhance NF‐κB and STAT3 signaling, further boosting the mechanisms for latency reversal.

TLR7 and TLR8, which are crucial sensors of SARS‐CoV‐2‐derived ssRNAs in macrophages, facilitate the release of TNFα, thereby indirectly promoting HIV latency reversal in J‐Lat cells [54, 55]. The interactions of SARS‐CoV‐2 with ACE2 in monocyte‐derived macrophages affect macrophage activation and cytokine release [17, 56]. Even with lower levels of pro‐inflammatory cytokines, such as TNFα, released from macrophages exposed to SARS‐CoV‐2 for 24 h, HIV latency reversal was still observed, suggesting that even transient M1 polarization may be sufficient [57]. In addition, growing evidence highlights that macrophage activation states cannot be fully captured by the classical binary M1/M2 framework. Single‐cell transcriptomic analyses and high‐dimensional phenotyping have revealed a spectrum of intermediate and mixed activation states, often characterized by coexpression of markers traditionally assigned to opposing polarization profiles. This heterogeneity reflects the dynamic plasticity of macrophages in response to complex microenvironmental cues, including viral infection and tissue‐specific signaling. Accordingly, future studies should adopt integrative approaches that go beyond categorical M1/M2 classifications to include multidimensional analyses of macrophage subsets, thereby providing a more accurate depiction of the functional diversity underlying SARS‐CoV‐2–driven immune modulation [8, 58].

The contrasting roles of TNFα and IL‐10 illustrate the complex relationship between macrophage polarization states and HIV latency. In the initial 24 h, M1 macrophages are predominant, releasing significant amounts of TNFα and fostering a pro‐inflammatory environment conducive to latency reversal. These macrophages also show a higher proportion of CD14^+^CD16^+^ cells, which are crucial in activating resting T cells. In laboratory settings, the interactions between CD14^+^CD16^+^ MDMs and T cells are critical for viral production [59]. As time progresses, extended exposure to SARS‐CoV‐2 leads to a shift in macrophage phenotype toward M2, characterized by elevated IL‐10 levels that dampen pro‐inflammatory signaling and help maintain latency [60]. This anti‐inflammatory transition may prevent excessive immune activation, acting as a protective mechanism while inhibiting the activation of viral reservoirs. Consistent with these findings, infliximab treatment, combined with cART and careful monitoring, may be both effective and well‐tolerated in patients with acute HIV infection [61–63]. Gaining insight into this balance could inform therapeutic approaches that leverage temporary pro‐inflammatory states while reducing the risks associated with chronic inflammation or immune suppression. The evolving understanding of macrophage heterogeneity in COVID‐19 highlights the need for more comprehensive phenotyping in future studies [58]. Finally, while our neutralization assays identified TNFα as the primary cytokine driving HIV latency reversal under the conditions tested, we cannot exclude contributions from additional soluble mediators released by SARS‐CoV‐2–exposed macrophages. Importantly, we did not perform experiments directly blocking TLR7/8 signaling, which could further substantiate the mechanistic link between viral RNA sensing and cytokine‐driven reversal of latency. Future studies employing multiplex cytokine profiling will enable a more comprehensive characterization of the inflammatory milieu associated with latency disruption. In contrast, combinatorial neutralization approaches targeting multiple cytokines simultaneously could delineate their relative or synergistic roles. Moreover, complementary strategies using TLR7/8 antagonists or inhibitory oligonucleotides, genetic silencing of TLR7/8, or pharmacological inhibition of endosomal acidification are warranted to rigorously test the causality of TLR7/8‐dependent pathways in the observed effects.

The absence of HIV reactivation with ROS inducers like TBH and rotenone, in contrast to the reactivation seen with PMA and TNFα, can be explained by the different pathways these agents activate. TBH induces oxidative stress that harms cellular components such as lipids, proteins, and DNA, activating stress‐response pathways like NRF2 and the unfolded protein response (UPR), but it does not trigger NF‐κB, which may be inhibited due to the extent of cellular damage. Additionally, excessive ROS might activate the DNA damage response (DDR), which can suppress HIV‐1 transcription, cellular antioxidant responses (like NRF2), and apoptosis, decreasing the number of viable cells available for reactivation studies.

This comparison shows that while ROS can influence certain pathways, their ability to trigger HIV reactivation varies by cell type, with oxidative stress possibly hindering rather than facilitating viral reactivation in lymphoid and monocytic models [12].

This study has several limitations. First, although the J‐Lat cell line is a well‐established model for studying HIV‐1 latency and reactivation, it does not fully reflect the biological complexity of resting CD4^+^ T cells—the main latent reservoir in vivo. While this model primarily captures early transcriptional activation via GFP expression under control of the HIV‐1 LTR, it remains a widely accepted tool for mechanistic studies. We acknowledge this limitation and emphasize that our findings provide insight into the initial steps of latency reversal induced by SARS‐CoV‐2‐stimulated macrophage signaling. Second, the in vitro system lacks the multicellular and extracellular microenvironment present in physiological tissues, limiting our ability to assess the contributions of cell‐to‐cell interactions, stromal components, and soluble mediators to HIV‐1 latency and immune modulation. Accordingly, while our findings offer mechanistic insights, they should be interpreted cautiously, and future studies employing ex vivo or in vivo models are warranted to validate and extend these observations. Regarding SARS‐CoV‐2 strain selection, we focused on the ancestral (Wh) and Omicron (BA.5) variants due to their clinical and temporal relevance. However, we acknowledge that other SARS‐CoV‐2 variants may exert differing effects on macrophage activation, and mutations—particularly in viral proteins involved in innate immune evasion—could influence the magnitude or nature of the observed responses. Therefore, further investigation using a broader range of variants is warranted to fully understand the spectrum of SARS‐CoV‐2–induced macrophage‐mediated effects on HIV latency.

In summary, this research indicates that SARS‐CoV‐2 does not directly cause HIV latency reversal in J‐Lat cells; instead, it does so indirectly by prompting the release of pro‐inflammatory cytokines, mainly TNFα, from macrophages exposed to SARS‐CoV‐2. These results underscore the intricate relationship between viral infections and the activation of HIV reservoirs. Future research should extend beyond the J‐Lat model to investigate the effects of SARS‐CoV‐2 on a wider array of HIV reservoirs, especially primary CD4^+^ T lymphocytes, to gain a deeper understanding of HIV reactivation dynamics in vivo. Additionally, examining methods to target cytokine signaling, such as TNFα, could provide valuable therapeutic options to prevent HIV reactivation in the context of coinfections like SARS‐CoV‐2. This approach may lead to the developing interventions that adjust the inflammatory environment to manage HIV reservoir activation without triggering detrimental immune responses.

## Author Contributions

Conceptualization, methodology, writing – review and editing: Patricio Jarmoluk, M. Victoria Delpino, and Jorge Quarleri. Software, validation: Patricio Jarmoluk, Franco Agustín Sviercz, and Cintia Cevallos. Formal analysis: Patricio Jarmoluk, and Cintia Cevallos. Investigation: Patricio Jarmoluk, Franco Agustín Sviercz, Cintia Cevallos, Rosa Nicole Freiberger, and Cynthia Alicia López. Resources: Jorge Quarleri and M. Victoria Delpino. Data curation: Patricio Jarmoluk. Writing – original draft preparation: Jorge Quarleri. Visualization: Patricio Jarmoluk, Rosa Nicole Freiberger, and Cynthia Alicia López. Supervision: M. Victoria Delpino and Jorge Quarleri. Project administration: Jorge Quarleri and M. Victoria Delpino. Funding acquisition: Jorge Quarleri and M. Victoria Delpino.

## Acknowledgments

AI was used for spelling checks and grammar corrections.

## Funding

This research was funded by the Agencia Nacional de Promoción Científica y Tecnológica (ANPCyT), PICTO‐2021‐00005‐COVID Secuelas (to Jorge Quarleri and M. Victoria Delpino) and by the Fundación Florencio Fiorini (to Jorge Quarleri, 2024).

## Disclosure

All authors agree to be accountable for the content of the work.

## Ethics Statement

The studies involving humans were approved by the Ethics Committee at the School of Medical Sciences (Universidad de Buenos Aires). The studies were conducted in accordance with the local legislation and institutional requirements. The participants provided their written informed consent to participate in this study (Number: RESCD‐2023‐872). Buffy coats from healthy donors, aged 18–60, with a balanced gender ratio, were sourced from Hospital de Clínicas “José de San Martín,” Facultad de Ciencias Médicas, Universidad de Buenos Aires. All human samples, obtained regardless of this study, were provided without personally identifiable information. This study was conducted following the local legislation and institutional requirements. No animal studies are presented in this manuscript. Inclusion of identifiable human data: No potentially identifiable images or data are presented in this study.

## Conflicts of Interest

The authors declare no conflicts of interest.

## Supporting Information

Additional supporting information can be found online in the Supporting Information section.

## Supporting information


**Supporting Information 1** Figure S2: Representative flow cytometry gating strategies used for immune cell phenotyping and HIV latency reversal assays. (A) Gating strategy for monocyte‐derived macrophages (MDMs). Live cells were selected based on SSC‐H vs. FSC‐H, followed by singlet gating (FSC‐H vs. FSC‐A) and exclusion of dead cells using Ghost Dye Violet450. CD14^+^ events were identified and further classified into M1 macrophages (CD80^+^CD206⁻) and M2 macrophages (CD80⁻CD206^+^). CD16 expression was also analyzed in combination with CD14 to identify nonclassical subsets. (B) Gating strategy for J‐Lat cells. Live cells were selected based on SSC‐A vs. FSC‐A. After viability and singlet gating, GFP expression was measured to assess HIV‐1 latency reversal. (C) Gating strategy for U1 cells. Live cells were selected based on SSC‐A vs. FSC‐A. Cells were fixed, permeabilized, and stained intracellularly with anti‐p24 antibody; HIV reactivation was quantified based on PE fluorescence intensity. All data were acquired using a Cytek Northern Lights 3000 flow cytometer and analyzed with FlowJo v10.6.2.

**Supporting Information 2** Figure S1: Relative J‐Lat cells death levels (%) measured by flow cytometry as indicated in M&M. (A) Cells exposed to cell‐free SARS‐CoV‐2 (Wh and BA.5 variants; MOI = 0.1). (B) Cells exposed to conditioned media from M1 and M2 polarized MDMs. (C) Cells were exposed to conditioned media from SARS‐CoV‐2‐infected MDMs for prolonged times (24, 48, and 72 h). (D) Cells were treated with TNF (10, 1, 0.1 ng/mL), and TNF was previously neutralized with infliximab (anti‐TNFα). PMA: C+ (positive control): J‐Lat cells exposed to freeze–thaw cycles. Data were obtained from 3 to 4 independent experiments and are presented as the mean ± SD, and statistical significance was calculated using one‐way ANOVA.

## Data Availability

The raw data supporting the conclusions of this article will be made available by the authors without undue reservation.
