# Supplementary figures and images for: SARS‐CoV‐2‐Induced Macrophage Polarization Reverses HIV‐1 Latency in J‐Lat Cells Through TNFα Signaling
Source: J Immunol Res. 2026 Feb 25;2026:9986845. doi: 10.1155/jimr/9986845 (PMC13140423; doi:10.1155/jimr/9986845)

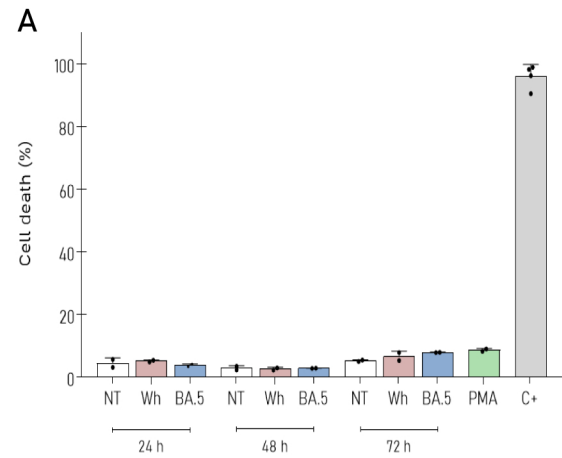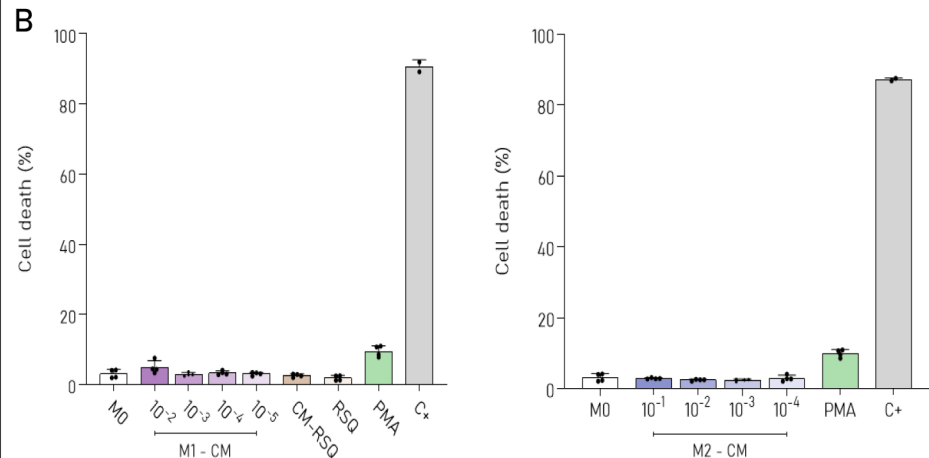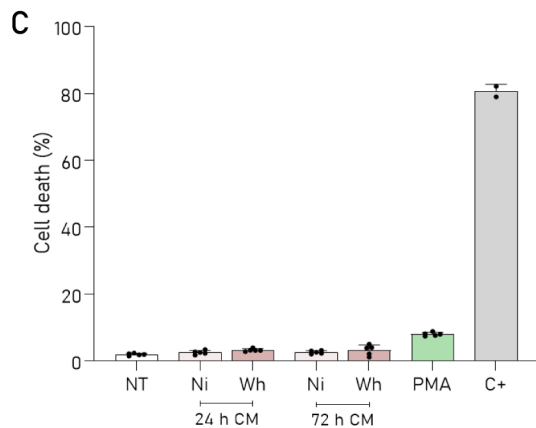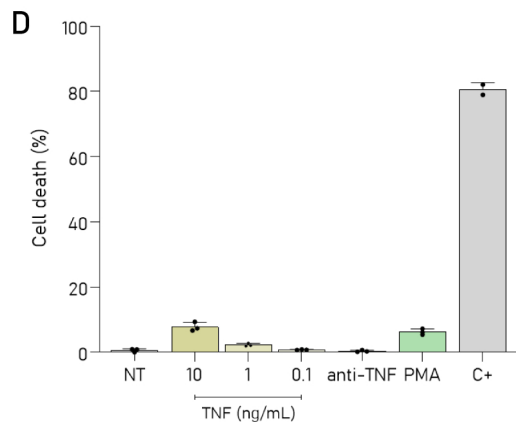

Supplement: Supplementary file 1 — Supporting Information 1 Figure S2: Representative flow cytometry gating strategies used for immune cell phenotyping and HIV latency reversal assays. (A) Gating strategy for monocyte‐derived macrophages (MDMs). Live cells were selected based on SSC‐H vs. FSC‐H, followed by singlet gating (FSC‐H vs. FSC‐A) and exclusion of dead cells using Ghost Dye Violet450. CD14+ events were identified and further classified into M1 macrophages (CD80+CD206⁻) and M2 macrophages (CD80⁻CD206+). CD16 expression was also analyzed in combination with CD14 to identify nonclassical subsets. (B) Gating strategy for J‐Lat cells. Live cells were selected based on SSC‐A vs. FSC‐A. After viability and singlet gating, GFP expression was measured to assess HIV‐1 latency reversal. (C) Gating strategy for U1 cells. Live cells were selected based on SSC‐A vs. FSC‐A. Cells were fixed, permeabilized, and stained intracellularly with anti‐p24 antibody; HIV reactivation was quantified based on PE fluorescence intensity. All data were acquired using a Cytek Northern Lights 3000 flow cytometer and analyzed with FlowJo v10.6.2. [file JIMR-2026-9986845-s002.pdf]

**A**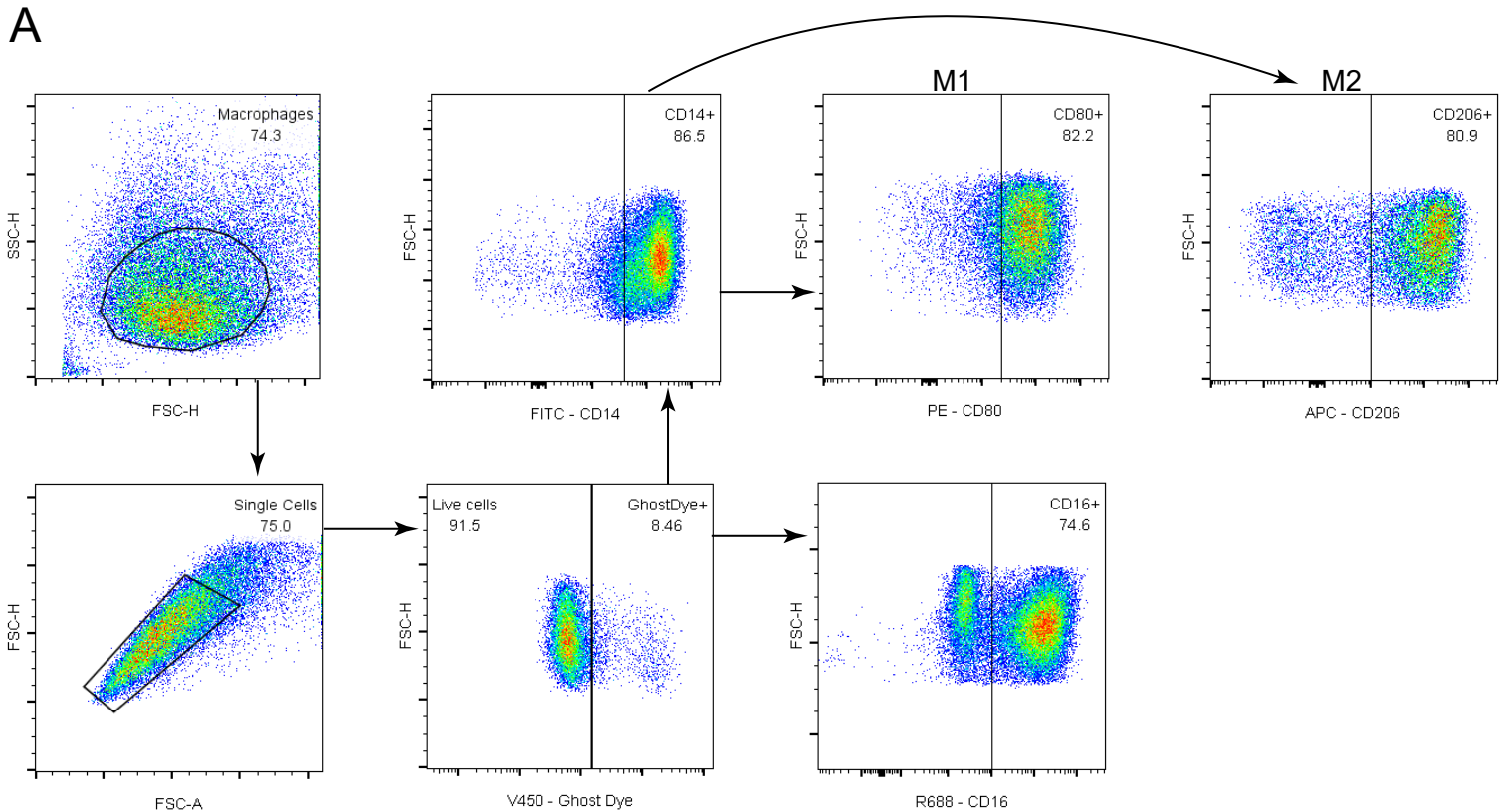**B**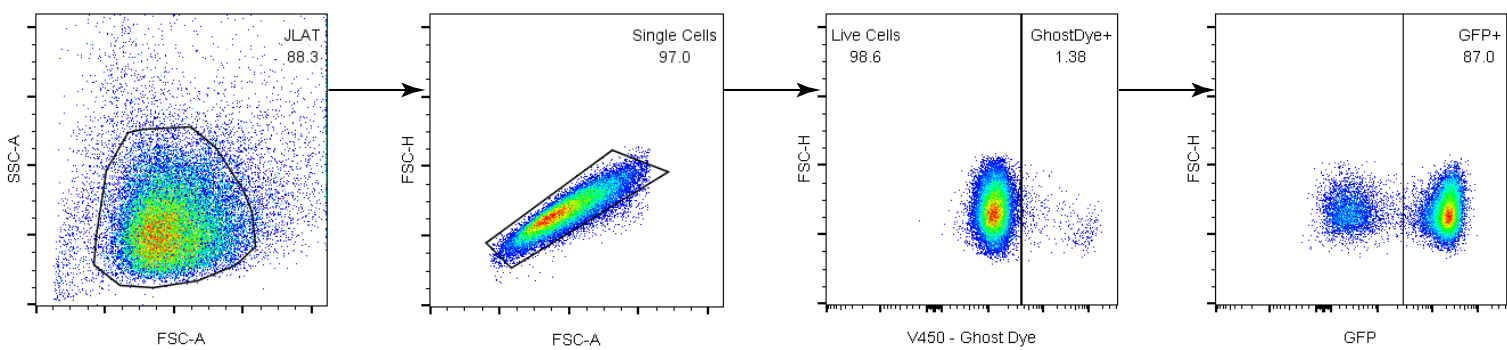**C**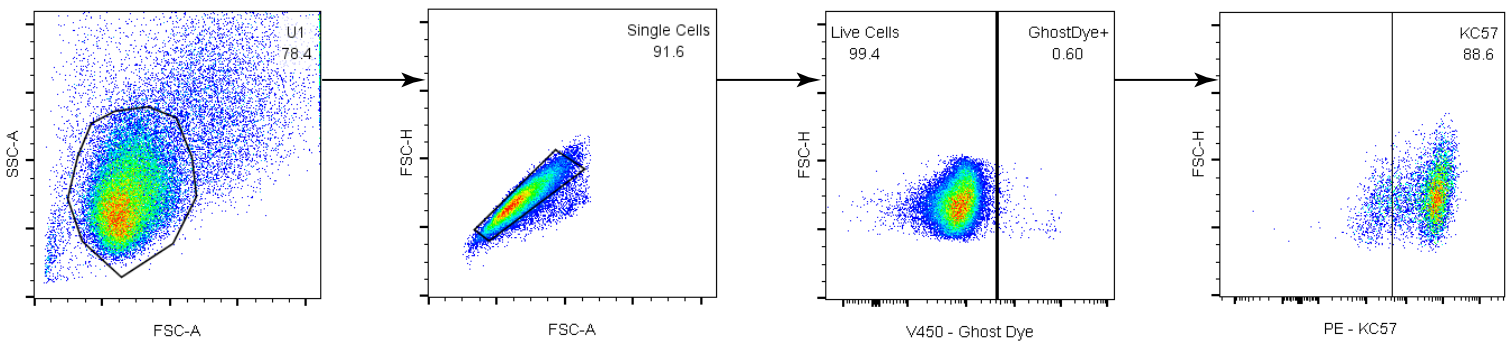

Supplement: Supplementary file 2 — Supporting Information 2 Figure S1: Relative J‐Lat cells death levels (%) measured by flow cytometry as indicated in M&M. (A) Cells exposed to cell‐free SARS‐CoV‐2 (Wh and BA.5 variants; MOI = 0.1). (B) Cells exposed to conditioned media from M1 and M2 polarized MDMs. (C) Cells were exposed to conditioned media from SARS‐CoV‐2‐infected MDMs for prolonged times (24, 48, and 72 h). (D) Cells were treated with TNF (10, 1, 0.1 ng/mL), and TNF was previously neutralized with infliximab (anti‐TNFα). PMA: C+ (positive control): J‐Lat cells exposed to freeze–thaw cycles. Data were obtained from 3 to 4 independent experiments and are presented as the mean ± SD, and statistical significance was calculated using one‐way ANOVA. [file JIMR-2026-9986845-s001.pdf]
